# Dental Pulp Stem Cell-Derived Organoids: Advancing the Development of 3D Structures

**DOI:** 10.3390/cells14201603

**Published:** 2025-10-15

**Authors:** Loreto Lancia, Fanny Pulcini, Emanuela Mari, Luca Piccoli, Leda Assunta Biordi, Luciano Mutti, Claudio Festuccia, Giovanni Luca Gravina, Vincenzo Mattei, Annunziata Mauro, Valentina Notarstefano, Simona Delle Monache

**Affiliations:** 1 Department of Biotechnological and Applied Clinical Sciences, University of L’Aquila, 67100 L’Aquila, Italy; loreto.lancia1@graduate.univaq.it (L.L.); fanny.pulcini@univaq.it (F.P.); assuntaleda.biordi@univaq.it (L.A.B.); luciano.mutti@univaq.it (L.M.); claudio.festuccia@univaq.it (C.F.); giovanniluca.gravina@univaq.it (G.L.G.); 2 Department of Life Science, Health, and Health Professions, Link Campus University, 00165 Rome, Italy; e.mari@unilink.it (E.M.); v.mattei@unilink.it (V.M.); 3 Department of Science Dentistry and Maxillofacial, Sapienza University of Rome, 00185 Rome, Italy; luca.piccoli@uniroma1.it; 4 Department of Bioscience and Technology for Food, Agriculture and Environment, University of Teramo, 64100 Teramo, Italy; amauro@unite.it (A.M.); vnotarstefano@unite.it (V.N.); 5 Center for Biotechnology, Sbarro Institute for Cancer Research and Molecular Medicine, College of Science and Technology, Temple University, Philadelphia, PA 19122, USA; 6 Division of Radiation Oncology, University of L’Aquila, 67100 L’Aquila, Italy

**Keywords:** organoids, mesenchymal stem/stromal cells, dental pulp stem cells, 3D models, cells differentiation

## Abstract

**Highlights:**

**What are the main findings?**

- The main aim of this article is to propose a validated and standardized protocol for characterising and conducting advanced structural analyses of dental pulp-like organoids using Raman microspectroscopy.

**What is the implication of the main findings?**

- These findings demonstrate improved reproducibility and reliability of these models and could have significant implications for their use in preclinical studies and drug screening.

- Furthermore, enabling Raman microspectroscopy to investigate the structural organisation of 3D dental pulp organoids paves the way for future clinical applications in the critical fields of bone tissue engineering and regenerative medicine.

**Abstract:**

Two-dimensional cell cultures are crucial research tools, and they have been widely used, although they are not completely representative of biological processes in vivo due to the lack of tissue architecture and complexity. Recent advances in organoid technology have addressed these limitations and are revolutionizing the tools available for in vitro culture. Although there are no unified protocols for generating organoids, they can be obtained with various techniques, leading to cell aggregation by promoting cell adhesion. This work aims to generate and characterise organoid models of dental pulp from dental pulp stem cells (DPSCs), a type of mesenchymal stem/stromal cells known for their high regenerative potential and ease of accessibility, to establish a model for translational studies. The organoids were subjected to osteogenic differentiation conditions. Cell viability was evaluated using a CCK-8 assay, while osteogenic morphology and mineralization were confirmed by Alizarin red analysis, Raman microspectroscopy, and by immunofluorescence for the lineage markers expression. The Alizarin red analysis indicated a higher presence of calcium phosphate deposits in the differentiated organoids than in the control group (CTR). These results were confirmed by spectral profiles obtained using Raman microspectroscopy, which were attributable to a hydroxyapatite-based biomaterial. Immunofluorescence analysis also revealed increased expression of odonto/osteogenic markers (RUNX and OSX), alongside reduced expression of stemness markers. In conclusion, the organoids appeared to have successfully differentiated into an osteogenic lineage, forming a mineralized matrix containing hydroxyapatite and showing increased expression of relevant lineage markers.

## 1. Introduction

In recent years, the advent and rapid evolution of 3D culture technologies-particularly organoid systems-have revolutionized in vitro culture tools for biomedical research overcoming the intrinsic limitation of traditional 2D cell culture systems. In this context, the advanced 3D platforms offer a more physiologically relevant microenvironment enabling the formation of self-organizing, organ-like structures that closely mimic the morphological and functional future of human tissues [1,2,3,4,5]. Today, organoids derived from pluripotent or adult stem cells can recapitulate key aspects of organogenesis and tissue-specific biology, making them invaluable tools for modelling human development, displaying a remarkable resemblance to their in vivo counterparts. Furthermore, human organoids are used to study models of infectious diseases, genetic diseases, and tumours and are obtained starting from different types of stem cells [3,6,7,8,9,10,11].

Within this contest, MSCs, one of the most studied types of ASCs, have attracted significant interest for their multipotency and regenerative capacity. Stem cells can be classified according to their fundamental properties and potency, i.e., the ability to differentiate into various cell types or lineages. Although the first studies conducted on MSCs have highlighted the possibility of isolating them from bone marrow, spleen, and thymus, it is now known that various other tissue districts are home to MSCs, such as collagen, periosteum, synovial fluid, muscles, and tendons. Moreover, there is increasing research on MSCs derived from alternative sources, including adipose tissue, peripheral blood, umbilical cord, and dental pulp [12,13]. The latter, DPSCs, can be easily isolated from discarded or removed teeth, especially from the third molar of young donors and are characterised by high regenerative potential [14].

Furthermore, DPSCs can differentiate into tissues of mesenchymal origin, including medullary stroma, adipose tissue, bone, cartilage, tendon, skeletal muscle, and visceral mesoderm. In addition, experimental evidence indicates that DPSCs can also differentiate into non-mesodermal cell types, such as neuronal cells, epithelial cells, endothelial cells, hepatocytes, renal cells, and lung cells [14,15]. For these reasons, DPSCs are excellent candidates for generating organoids and, therefore, to obtain experimental dental pulp organoids models.

In this contest, 3D organoid-based technology is increasingly being used in tissue engineering for many organ types and drug screening [16,17,18,19,20,21,22] and recently, dental pulp cells, tissues and organoids have also been considered as models for toxicity assessment of materials/drugs [21,22,23,24].

Several studies have attempted to generate dental pulp-like organoids or spheres [19,20,21] demonstrating that both have potential for dental tissue regeneration. The protocols developed for this purpose propose different experimental strategies, based on different culture conditions, either in the presence or absence of additional cell types and often combined with scaffold, 3D bioprinting technologies or microfluidic devices [19,20,21,22,23,24].

The development of innovative characterisation strategies for 3D models has the potential to offer a distinct advantage by more effectively demonstrating the extent to which the tissue and microenvironment of organoids are replicated. Despite these advances, there are still many methodological challenges to resolve, such as reproducibility, standardization, costs, and 3D structural analysis of odonto/osteogenic characteristics.

Most standard approaches for determining gene and protein expression profiles, such as RT-qPCR and Western blotting, require complete tissue lysis by disrupting the 3D architecture of the organoid. This is not always easy, especially in the presence of the mineralized tissue. In addition, morphological techniques based on histological staining or immunofluorescence face issues result in incomplete or inaccurate representations of internal cellular environment, particularly in dense or mineralized tissues such as those found in dental pulp organoids due to limited penetration of dyes, antibodies, or other labelling reagents into the deeper layers of the organoids.

Therefore, the aim of our work has been to generate dental pulp organoids with high potential for bone tissue engineering and regenerative applications, by employing a validated, streamlined, and reproducible guided protocol [20]. This approach offers an efficient alternative to more complex methodologies, facilitating broader adoption in research and clinical settings.

To evaluate the effectiveness of the proposed protocol, we investigated the structural organization of the resulting 3D dental pulp organoids using advanced analytical techniques including 3D immunofluorescence, and Raman microspectroscopy [25]. Raman microspectroscopy offers a powerful, non-invasive alternative that complements traditional methods by providing high-resolution, spatially informative, and label-free analysis. The integration of these techniques enhances the overall characterisation of organoids, offering deeper insights into their architecture and functionality [26,27].

## 2. Materials and Methods

### 2.1. Primary Cell Lines and Cultures

DPSCs were obtained from the impacted third molar of young adult patients (13–19 years of age), affected by dysodontiasis, for whom the third molar represented discarded material and was thus easily accessible for research purposes. These samples were collected with the informed consent of the patients, in accordance with ethical considerations, after signing the appropriate forms, and with the approval of the ethics committee of the Umberto I Polyclinic of Rome (Project identification code: 4336). The patients were treated at the Department of Science, Dentistry, and Maxillofacial at Sapienza University of Rome.

DPSCs were maintained in DMEM-LG, supplemented with 100 units/mL penicillin, 10 mg/mL streptomycin, and 10% FBS (Euroclone, Milan, Italy). The cells were maintained in culture in a humidified environment with 5% CO_2_ at 37 °C and then characterised by flow cytometry for mesenchymal stromal cells (MSCs) expression according to the minimal criteria defined by the International Society for Cellular Therapy (ISCT) and to our previous studies [12,28,29]. For the subsequent experiment, cells were used between the 2nd and 5th passage.

### 2.2. Development of Organoids from Dental Pulp Stem Cells

Organoids were generated according to the protocol described by Lancia et al. [20]. Briefly, three-dimensional structures were created using DPSCs at passage 4 using a stationary technique. Initially, 10 μL droplets of Matrigel^®^ Matrix (Corning, New York, NY, USA) containing 75,000 cells were prepared and allowed to polymerize for 30 min at 37 °C. The polymerized droplets were then transferred to a 96-well U-bottom plate (Sarstedt AG & Co. KG, Numbrecht, Germany), and cell aggregation was monitored using a Leica Mateo TL microscope (Leica Biosystems, Milan, Italy).

### 2.3. Osteogenic Differentiation of Organoids

**Scheme 1:**
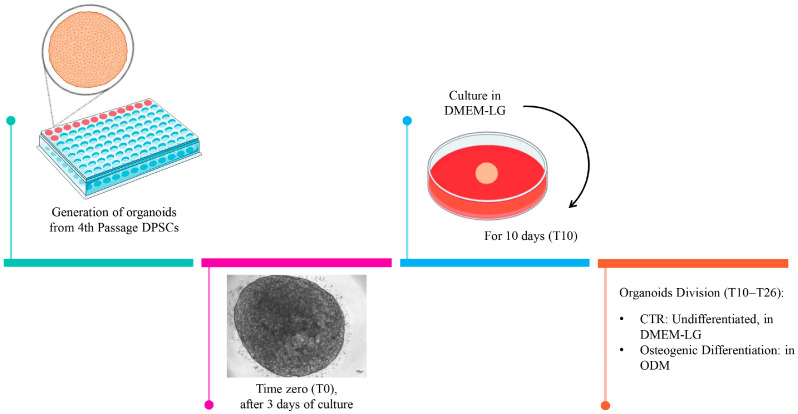
Representative schematic steps for organoid growth. Following the generation of organoids, after three days, adequate cell aggregation was observed, leading us to consider this as our time zero (T0). After 10 days (T10) of culture, the organoids were divided into two groups: undifferentiated, maintained in DMEM-LG into the 96-well U-bottom, and the other group subjected to osteogenic differentiation with specific culture medium (ODM) from T10 to T26.

Osteogenic differentiation was induced by cultivating the 3D structures of DPSCs in a specific osteogenic differentiation medium (ODM) consisting of a basal medium (DMEM-LG) supplemented with 50 mM ascorbic acid, 10 mM β-glicerophosphate, 0.1 µM dexamethasone, and 5% FBS for 26 days, being refreshed every 2–3 days (200 µL/well). The cultures were carried out in parallel with the controls, which were maintained in standard medium (DMEM-LG) under the same conditions and at the same times, as shown in Scheme 1.

### 2.4. Organoids Viability Evaluation

To assess organoid viability, a colorimetric cell counting kit-8 assay (Sigma-Aldrich, St. Louis, MO, USA) was used according to manufacturer instructions. Briefly, 10 µL of CCK-8 reagents were added to each well of a 96-well U-bottom plate containing the organoids and 100 µL of culture medium. After a 4-hour incubation at 37 °C, absorbance at 450 nm was measured using a microplate reader (Infinite M200 Pro, Tecan, Nänikon, Switzerland). The absorbance value is directly proportional to the amount of formazan dye produced by viable cells, thus reflecting their viability.

### 2.5. Organoids Morphological Evaluation by Stereo Microscope

The Zeiss Axio Zoom v6 (Zeiss, Oberkochen, Germany) stereo microscope proved invaluable for the detailed morphological analysis of organoids. Its ability to capture high-resolution, three-dimensional images through Z-stacking allowed for comprehensive visualization and in-depth study of the morphology of these complex models. Before imaging, organoids were gently washed in PBS to remove any residual media or debris, ensuring optimal clarity during observation. Subsequently, organoids were carefully placed on a plate and positioned under the Axio Zoom v6 for examination. High-quality images were acquired at a magnification of 63×.

### 2.6. Preparation of Frozen Sections and Hematoxylin and Eosin Staining

The organoids were washed in PBS and fixed in 4% PFA (Santa Cruz Biotechnology, Dallas, TX, USA) for 30 min at 4 °C. After a brief washing in PBS, the organoids were embedded in OCT embedding medium for frozen tissues (Scigen, Scigen Scientific, Gardena, CA, USA) by moulds, and rapid freezing in nitrogen was performed by adding a cryoprotectant (FBS-DMSO 9:1). Next, they were cryosectioned using a Leica CM1859 cryostat (Leica Biosystems, Milan, Italy) to obtain 14 µm-thick sections. The sections obtained were mounted on Superfrost Plus slides (Menzel-Gläser, Braunschweig, Germany) and stored at −80 °C until use. The sections were then left at RT for a few minutes and, subsequently, were used for hematoxylin and eosin staining. The slides were subsequently assembled with a coverslip using glycerol as a mounting medium. A microscope, Leica Mateo TL (Leica Biosystems, Milan, Italy), was used to analyze the samples.

### 2.7. Alizarin Red Staining

The sections obtained, as described in Section 2.6, were stained using Alizarin red. First, the slides were washed in PBS and then fixed with 4% PFA (Santa Cruz Biotechnology, Dallas, TX, USA) at RT for 10 min. After three washes in PBS, the samples were incubated with 2% Alizarin red (Sigma-Aldrich, Milan, Italy) for 10 min and rewashed with PBS to remove excess stains. The dehydration of the samples was performed using a graded ethanol series (70% for 3 min, 95% for 3 min, and 100% for 3 min).

After the dehydration, the slides were mounted using glycerol. Staining images were captured using a Leica Mateo TL microscope (Leica Biosystems, Milan, Italy).

### 2.8. Immunofluorescence Analysis

The samples were cryosectioned as described in Section 2.6, and also used for immunofluorescence analysis. The slides were washed twice for 5 min with PBS to remove OCT residue. To reduce autofluorescence and eliminate excess Matrigel^®^ Matrix, the sections were incubated in a commercially available Cell Recovery Solution (Corning, New York, NY, USA) for 20 min at 4 °C.

At this point, the samples were permeabilized with 0.05% Triton X-100 (Thermo Fisher Scientific, Waltham, MA, USA) in PBS for 10 min at 4 °C, followed by three 5 min washes in PBS. Non-specific binding was blocked using a 3% BSA solution for 1 hour at RT. Then, samples were incubated with the following primary antibodies:

- CD105, monoclonal, mouse (Santa Cruz Biotechnology, Dallas, TX, USA), dilution 1:50
- OSX monoclonal, mouse (Santa Cruz Biotechnology, Dallas, TX, USA), dilution 1:50.
- RUNX2, monoclonal rabbit (Cell Signaling Technology Inc., Danvers, MA, USA), dilution 1:250.
- FITC-Phalloidin mouse (Merk Life Science, Sigma Aldrich, Milan, Italy), dilution 1:1000.

All primary antibodies were diluted in a blocking buffer to reduce any background and were incubated overnight at 4 °C protected from light. The slides were washed in PBS for 15 min (3 washes/5 min per wash) and lightly dried at RT. The samples were treated with an anti-mouse secondary antibody labelled with Alexa fluor 488 and anti-rabbit secondary antibody labelled with Alexa Fluor 594 (ThermoFisher Scientific, Rockford, IL, USA), used at a 1:1000 dilution in PBS and incubated for 1 h at RT, on the dark. The slides were then washed in PBS for 15 min (3 washes/5 min per wash) and lightly dried again; for nuclei staining, DAPI (Invitrogen, Thermo Fisher Scientific, Waltham, MA, USA) diluted 1:1000 in PBS was added to each slide. Next, the samples were washed in PBS for 15 min (3 washes/5 min per wash), dried, and mounted with a coverslip using glycerol as a mounting medium. The samples were analyzed, and images were acquired with the A1 Zeiss Axiovert fluorescence microscope (Zeiss, Oberkochen, Germany).

### 2.9. Raman Microspectroscopy (RMS)

A Horiba Jobin-Yvon XploRA Nano Raman Microspectrometer, equipped with a 785 nm diode laser was used as a source. All RMS measurements were performed by using a ×100 objective (Olympus, Tokyo, Japan). Prior to spectral acquisition, the spectrometer was calibrated to the 520.7 cm^−1^ line of silicon. A 1200 lines per mm grating was chosen. A 200 mm confocal pinhole was used for all measurements. The spectra were dispersed onto a 16-bit dynamic range Peltier cooled CCD detector. For each experimental group, n. 10 Raman spectra were collected n. 3 decellularized specimens. Organoids were decellularized with minor modifications to the protocol of Li et al. [30]. Briefly, organoids were incubated in 0.025% (*w*/*v*) trypsin-EDTA for 90 min at 37 °C. They were then treated with 5% (*v*/*v*) Triton X-100 for 120 min at RT with agitation (120 rpm). After PBS washes, samples were fixed in 4% PFA for 24 h at 4 °C, washed again in PBS and stored at 4 °C until Raman spectroscopy analysis. All Raman spectra were corrected for the contribution of atmospheric carbon dioxide and water vapour (Atmospheric compensation routine, OPUS 7.5 software, Bruker Optics, Ettlingen, Germany) and vector normalized in the full spectral range (Normalization routine, OPUS 7.5 software). Then, specific area, intensity, and FWHM (full width at half maximum) values were calculated to assess well-defined spectral markers (Integration routines, OPUS 7.5 software).

### 2.10. Statistical Analysis

Data are reported as mean ± SD based on three independent experimental replicates (*n* = 3 per treatment and time point). Statistical analyses were performed using Prism 6 software (GraphPad Software, La Jolla, CA, USA). Group comparisons were conducted using Student’s *t*-test or one-way ANOVA, as appropriate. A *p* ≤ 0.05 was considered indicative of statistical significance.

## 3. Results

### 3.1. Establishment of 3D Organoids Cultures

DPSCs were isolated from third molars of young donors, cultured, and grown in DMEM-LG under controlled temperature and atmosphere conditions as reported in Scheme 1. Preliminary experiments confirmed the expression of MSCs markers and their adherence to plastic in culture. Starting from T0, the DPSCs aggregation was monitored evaluating the increase in cell density through time-lapse imaging. Over time, a progressive increase in cell density was observed in the formed structures, reaching an approximate diameter of 1.5 mm after 26 days of maintenance in culture. To evaluate the differentiation capacity of the organoids, at T10 we replaced the medium with the specific differentiation medium (ODM).

**Figure 1:**
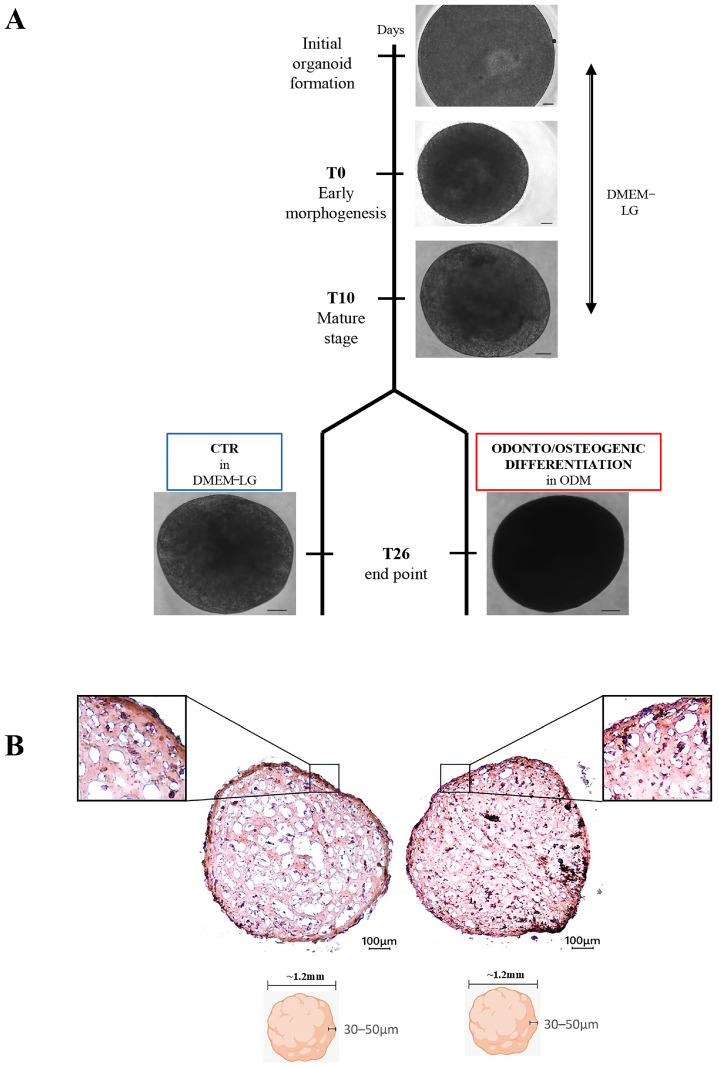
Generation of organoids. (**A**) From T0 to T26 end point. Representative images of development of organoids maintained in DMEM-LG (CTR) and organoids undergoing odonto/osteogenic differentiation (ODONTO/OSTEO) acquired using the optical Leica Mateo TL microscope (Magnify 4×). The scales correspond to 200 μm. (**B**) Representative images of the hematoxylin and eosin staining of the CTR and ODONTO/OSTEO organoids acquired using the optical Leica Mateo TL microscope (Magnify 10×). The scale correspond to 100 μm.

The samples were divided into two groups as specified above (Figure 1), and their development was followed through a Leica Mateo TL microscope (Leica Biosystems, Milan, Italy).

As shown in Figure 1A, an increase in the cell density (as highlighted by the apparent darker colour of the organoid itself) of the differentiated organoids was observed compared to CTR organoids. Organoid sections were then used to investigate their internal structure and morphology. Using hematoxylin and eosin staining, it was possible to observe at the endpoint (T26) a well-compacted area with cells nuclei (blue stain) distributed in the outermost part of structure, in contrast to the innermost which appeared disorganized and loose with few cells (Figure 1B). In particular, the thickness from the outside was about 30 μm in both the differentiated and control organoids (Figure 1B). This aspect was probably due to a higher cell mortality rate caused by a reduced supply of oxygen and nutrients in the internal structure (core) of the organoids. This phenomenon could be due to a higher cell mortality rate caused by a reduced supply of oxygen and nutrients in the internal structure (core) of the organoid [3,31].

### 3.2. Organoids Vitality

**Figure 2:**
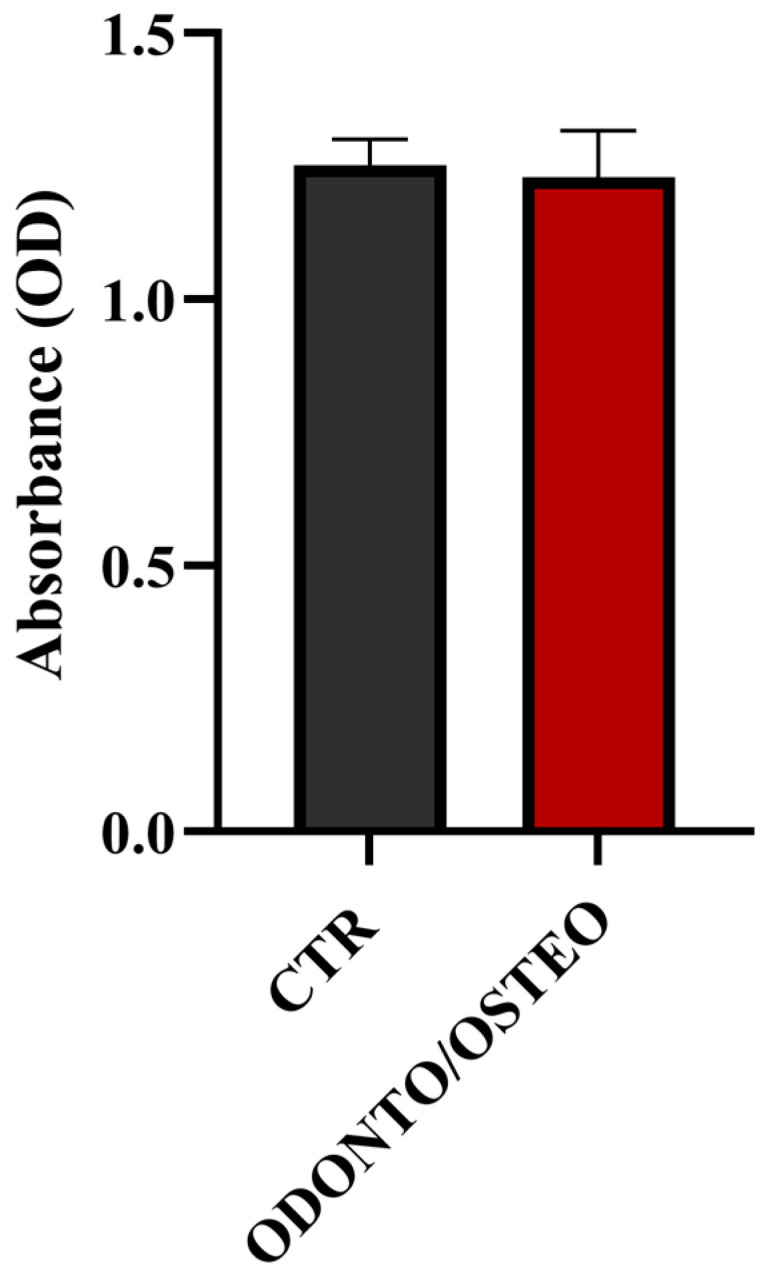
Cell proliferation assays of organoids derived from dental pulp cells. Proliferation rates were conducted on both CTR organoids (CTR) and differentiated organoids (ODONTO/OSTEO) at the end of differentiation protocol (T26). Seven independent experimental replicates/treatments (*n* = 7) were performed.

DPSCs cultured under basal conditions (CTR) or induced to differentiate (ODONTO/OSTEO) were assessed for the cell proliferation rate. The CCK-8 assay, which measures cellular metabolic activity associated with proliferation, shows no statistically significant differences between the CTR and ODONTO/OSTEO groups (Figure 2), although morphological observation showed an increase in the apparent density of differentiated organoids. This suggested that the dark staining observed under the microscope was associated with the presence of a matrix, and that the cell density in differentiated organoids of the same size was lower than in control organoids.

### 3.3. Morphological Features of Dental Pulp Organoids

**Figure 3:**
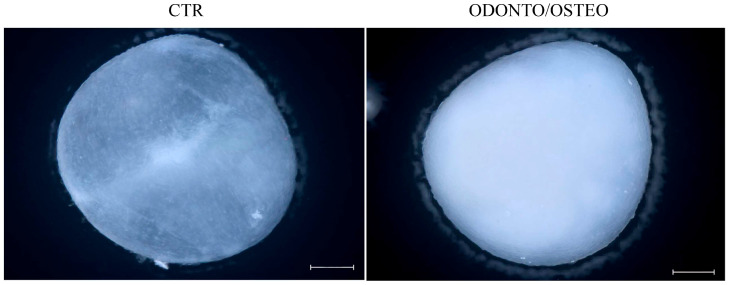
Stereomicroscopic analysis of DPSC-derived organoids cultured in control and differentiation conditions. Representative images of organoids after 26 days in standard culture medium (CTR) and osteogenic differentiation medium (ODONTO/OSTEO), acquired using a Zeiss Axio Zoom.v16 stereo microscope (Magnify 63×). The scales correspond to 200 μm.

Organoids of the control (CTR) and differentiated (ODONTO/OSTEO) derived from DPSCs were observed by the stereo Zeiss Axio Zoom.v16 microscope (Zeiss, Oberkochen, Germany). Figure 3 shows that CTR organoids exhibited a regular spherical morphology with a smooth, translucent, and homogeneous surface. No macroscopic evidence of mineralization was observed. Conversely, ODONTO/OSTEO organoids displayed a more irregular surface and a more opaque and white appearance (Figure 3).

### 3.4. Calcium Carbonate Deposition in Dental Pulp Organoids

**Figure 4:**
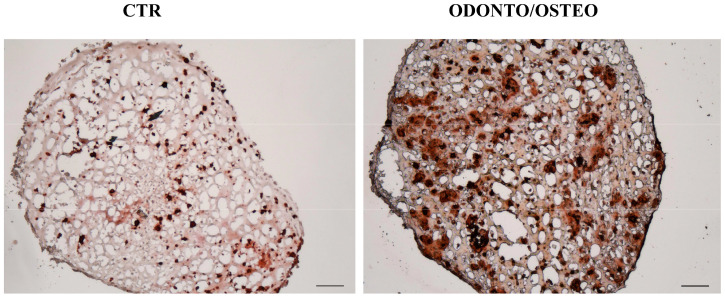
Calcium carbonate deposition in DPSC organoids. Alizarin red staining of control (CTR) and differentiated (ODONTO/OSTEO) organoids. Scale bar of 100 μm (4× magnification).

An extensive and intense mineralization was observed in cryosections of (ODONTO/OSTEO) differentiated organoids in Figure 4 compared to CTR. A significant increase in calcium salt deposition after Alizarin red stain demonstrates a mineralized dental tissue formation, suggesting that the differentiated pulp cells (ODONTO/OSTEO) have acquired a mature odonto/osteogenic-like phenotype capable of an effective extracellular matrix mineralization. In contrast, the CTR group showed no significant calcium accumulation. These results, which showed strong positive calcium deposition in the differentiated organoids, confirmed morphological and chromatic changes observed in previous results.

### 3.5. Characterisation of Chemical Structure and Crystallinity by RMS

**Figure 5:**
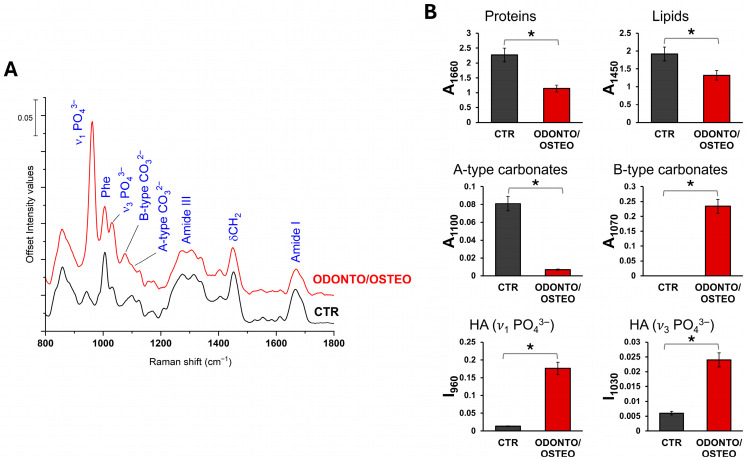
Raman microspectroscopy analysis of CTR and ODONTO/OSTEO organoids. (**A**) Average Raman spectra representative of CTR and ODONTO/OSTEO experimental groups. Labels highlight the peaks of interest; for better viewing, spectra are offset along the *y*-axis. (**B**) Statistical analysis of the numerical variation in proteins, lipids, A-type carbonates, B-type carbonates, and HA (ν_1_ and ν_3_) calculated values. Three specimens (*n* = 3) were analysed from each group, with ten spectra obtained from each specimen. Data are presented as mean ± SD. * Statistically significant value of ODONTO/OSTEO box charts with respect to CTR (*p* < 0.05; Student’s *t*-test).

Figure 5A reports representative Raman spectra of CTR and ODONTO/OSTEO experimental groups, and the behaviour of the most significant peaks is highlighted in Figure 5B: proteins (area of the band centred at 1660 cm^−1^, Amide I peak of proteins) [32,33]; lipids (area of the band centred at 1450 cm^−1^, bending of CH_2_ moieties, mainly from aliphatic chains) [25,34]; A-type carbonates (area of the band centred at 1100 cm^−1^, A-type CO_3_^2–^ groups) [32]; B-type carbonates (area of the band centred at 1070 cm^−1^, B-type CO_3_^2–^ groups) [25,33]; hydroxyapatite (HA) (intensity of the band centred at 960 cm^−1^, first stretching mode (ν_1_) of PO_4_^3−^ groups of HA) [26,34]; and hydroxyapatite HA (intensity of the band centred at 1030 cm^−1^, third stretching mode (ν_3_) of PO_4_^3−^ groups of HA) [25,34]. The evaluation of these peaks highlights that, especially post-differentiation, Raman spectra acquired features typical of bioapatite (HA), displaying all the characteristics peaks, including those related to carbonates and phosphates; for all the selected spectral markers, significant changes were found between CTR and ODONTO/OSTEO groups. As regards the significantly decreased content in proteins and lipids, it is clearly ascribable to an increased relative content of mineral deposition, possibly in the form of HA. In general, the comparison of Raman spectra from the two experimental groups displays a shift from almost all A-type CO_3_^2−^ moieties in CTR to almost all B-type CO_3_^2−^ in ODONTO/OSTEO.

**Figure 6:**
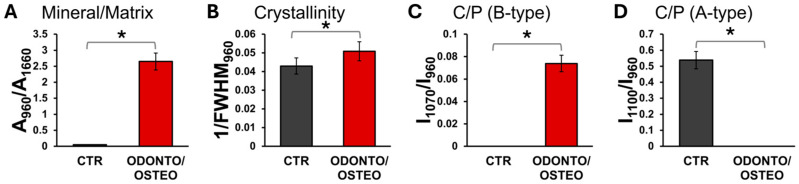
Raman spectral markers of differentiation. Statistical analysis of the numerical variation in (**A**) mineral/matrix, (**B**) crystallinity, (**C**) C/P (B-type), and (**D**) C/P (A-type) spectral markers. Three specimens (*n* = 3) were analysed from each group, with ten spectra obtained from each specimen. Data are presented as mean ± SD. * Statistically significant values of ODONTO/OSTEO box charts with respect to CTR. (*p* < 0.05; Student’s *t*-test).

In addition, the three widely employed spectral markers mineral/matrix (A_960_/A_1660_), crystallinity (1/FWHM_960_), and C/P (carbonates/phosphates, I_1070_/I_960_) were calculated and compared between CTR and ODONTO/OSTEO (Figure 6).

### 3.6. Immunofluorescence Analysis of DPSCs Organoids

**Figure 7:**
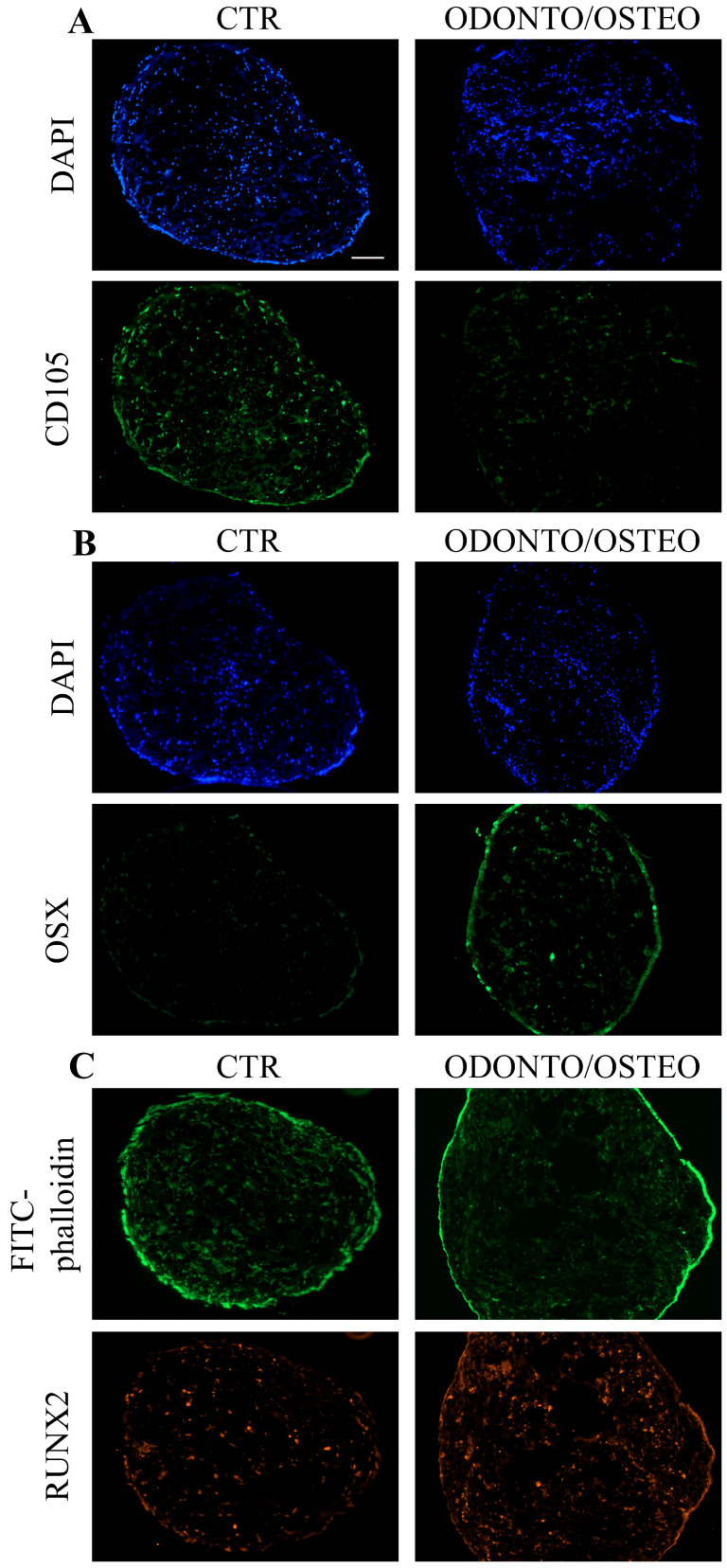
Representative images of immunofluorescence analysis of organoids for stemness and differentiation (ODONTO/OSTEO) markers. (**A**) Images show expression of CD105 in the CTR and differentiated organoids. (**B**) Images show expression of specific ODONTO/OSTEO markers (OSX) in CTR and differentiated organoids. (**C**) Images represent RUNX2 expression in CTR and differentiated samples. The samples were analyzed, and images were acquired with the A1 Zeiss Axiovert fluorescence microscope (Zeiss, Oberkochen, Germany). The scales correspond to 200 μm (10× magnification).

The expression markers of odonto/osteogenic differentiation (RUNX2 and OSX) and the marker of stemness (CD105) (Figure 7) were evaluated in cryosections of both organoids’ groups by immunofluorescence analysis. A solid fluorescent signal that accumulated due to the various focal planes was observed in the slices. Furthermore, the shallow depth of field of the high magnification objectives did not allow us to obtain good-quality images; despite this, we could observe the expression of the various markers of differentiation and stemness and perform a comparison between the undifferentiated and differentiated organoids.

Images obtained under a fluorescence microscope showed in the control organoids a higher expression of stem marker CD105 in comparison to differentiated organoids (green-Figure 7A). Moreover, we observed a low expression of specific odonto/osteogenic markers (OSX) (green-Figure 7B). Conversely, in differentiated organoids, we observed an apparent reduction in the expression of the CD105 (green-Figure 7A) and an increase in OSX and RUNX2 expression, which is typically greater in DPSCs differentiated versus undifferentiated samples (Figure 7B, C).

## 4. Discussion

This study allows the development and characterisation of a 3D model of DPSCs organoids using a relatively simple guided protocol previously established [20]. Our goal was to evaluate the ability to generate odonto/osteogenic organoids and also to explore the effectiveness of alternative methods to analyze their structural organization, including 3D immunofluorescence and Raman microspectroscopy, the latest technique capable of providing a fingerprint of the organoid differentiation state [32]. Indeed, the integration of these techniques is ideal to offer a combined functional and structural perspective of organoid development in terms of matrix composition and mineral deposition. In particular, our results showed that the calcium phosphate deposition was higher in differentiated organoids (after 16 days of differentiation) than in the control group. In parallel, Raman microspectroscopy revealed spectral profiles consistent with those expected for a hydroxyapatite-based biomaterial. Furthermore, immunofluorescence analysis of the differentiated organoids revealed the enhanced expression of odonto/osteogenic markers (RUNX2 and OSX), as well as reduced expression of the stemness marker CD105. This confirms the observed morphological changes.

After 26 days in culture, our protocol yielded organoids with a diameter of around 1.5 mm. This result was like to those achieved by other researchers, who utilized different and/or more sophisticated techniques involving the mixing of DPSCs with EC with or without hDP-ECM [19,24]. Throughout the culture period, we observed a progressive increase in microscopically visible cell density, although results from the CCK-8 assay, which measures cellular metabolic activity, did not show statistically significant differences between the CTR and ODONTO/OSTEO groups of organoids. Recently, we have demonstrated by trypan blue exclusion staining assay that the number of viable cells in ODONTO/OSTEO organoids was reduced with respect to CTR [20]. As reported in several reports, it is likely that the metabolic activity of cells during differentiation does not always correlate linearly with cell proliferation [20]. The histological analysis of cryosectioned samples showed a more precise organisation of the cells on the outer layer and, on the contrary, a more disorganised inner structure, especially in the central region. This spatial heterogeneity is a well-documented phenomenon in 3D cell culture systems, particularly in 3D structures exceeding 500 µm in diameter. In these cases, the innermost cells become increasingly distant from the culture medium, preventing efficient diffusion of oxygen and nutrients [3,31]. These preliminary results were corroborated by macroscopic observation of the morphological differences between CTR and differentiated organoids. In particular, the first appeared smooth and translucent while differentiated organoids showed an opaque surface. These features can be in line with the presence of mineralized content and phenotypic changes induced by differentiation. In fact, the presence of the functional calcium salt deposition confirmed a substantial presence in the differentiated organoids of a mineralized extracellular matrix. These results reinforce the known ability of DPSCs to acquire an odontogenic/osteogenic-like phenotype in vitro, consistent with previous studies demonstrating their potential to form mineralized tissue [14,35,36].

Then, Raman microspectroscopy was employed to provide an accurate chemical identification and characterisation of the molecular structures of the obtained organoids, investigating both organic and inorganic components. This technique provides accurate and precise spectral information about the minerals present by examining, in a label-free way, the characteristic energies of their vibrational modes [35]. Notably, the spectra of ODONTO/OSTEO samples exhibited all the characteristic signatures of biological apatite including intense and well-defined phosphate (PO_4_^3−^) bands at 960 cm^−1^ (ν_1_) and 1030 cm^−1^ (ν_3_), accompanied by signals corresponding to carbonate substitutions. As previously described, two types of carbonate groups have been found and are reported to exist in dental mineral material: A-type (1100 cm^−1^) and B-type (1070 cm^−1^); in particular, the latter represents the 1 symmetric stretching mode of CO_3_^2−^ ions replacing PO_4_^3−^ in the apatite lattice [34,35,36], while A-type carbonates substitute OH- moieties [34]. This carbonate substitution has been reported to be critical in HA’s solubility; in fact, changes in the intensity of these bands have been found in the spectroscopic characterisation of carious lesions [36]. Moreover, the three spectral markers mineral/matrix (A_960_/A_1660_), crystallinity (1/FWHM_960_), and C/P (carbonates/phosphates, I_1070_/I_960_) were calculated and compared. The mineral/matrix ratio reflects the relative amount of the mineral component with respect to the organic one, using the n1 symmetric stretching mode of PO_4_^3−^ (960 cm^−1^), being the most intense HA mineral band, and Amide I (1660 cm^−1^), representing all proteins [34]. The significantly higher value found in ODONTO/OSTEO samples may reflect the deposition of a more mature mineral dental material.

In general, the FWHM of Raman peaks is an indicator for crystalline quality; in this paper, to assess HA’s Crystallinity, the inverse of FWHM960 is used, meaning that narrow band widths are related to a high mineral crystallinity and vice versa [35]. As shown by the histograms in Figure 6B, although significant, the difference in crystallinity between CTR and ODONTO/OSTEO is not as striking as other investigated parameters: this is clearly attributable to the deeply different shape and height of the examined peak in the two sets of spectra, with the CTR one being very low and, hence, poorly comparable with the ODONTO/OSTEO.

The C/P ratio is calculated as the ratio between the intensities of the peaks at 1070 or 1100 cm^−1^ (B- or A-type) and at 960 cm^−1^, respectively, assigned to carbonate and phosphate groups in HA. In the literature, a progressive increase in this ratio (mainly the one referred to B-type carbonates) is reported to be related to a demineralization process, like the one occurring in caries [37]. In this case, the behaviour of the C/P ratio appears deeply different when considering A-type or B-type carbonate groups: here, the results only suggest a very different crystal structure between CTR and ODONTO/OSTEO.

In addition, the successful differentiation of DPSC-derived organoids into the odonto/osteogenic lineage was confirmed. CTR organoids showed high expression of the stem cell marker CD105 and low levels of differentiation markers RUNX2 and OSX. In contrast, ODONTO/OSTEO organoids, consistent with previous studies, showed reduced CD105 and increased RUNX2 and OSX expression [38]. Although the quality of the images was partially limited by the thickness of the section, the depth of field of the microscope, and autofluorescence problems related to the presence of the matrix, the differences in the expression of the markers were clearly visible [39].

The limitation of our study is the small number of “n” samples investigated. This is due to the difficulty of producing a large number of organoids by manual method. Conversely, standardizing organoid production using innovative automated approaches requires expensive equipment. Furthermore, the relatively brief organoid culture time of 26 days could represent a limitation, which we propose to overcome in future studies by culturing organoids for up to 1–2 months. However, this approach could exacerbate the limitations associated with the use of a stationary culture technique that leads to the formation of a necrotic core as the organoids increase in size over time.

Another limitation is the small number of osteogenic and stemness markers investigated. We plan to expand the range of markers used in the future to include early and late markers. This will help to improve our understanding of the temporal dynamics of osteogenic differentiation over time. This will be performed in conjunction with Raman microspectroscopy.

In conclusion, although further studies over longer time windows and intermediate time frames could be conducted to evaluate the possibility of obtaining mature tooth/bone mineral material, all these results, along with others, validate our differentiation protocol and highlight the potential of 3D organoid models for studying DPSC biology and lineage commitment.

## Data Availability

The data presented in this study are available in the article. Row data are available on request from the corresponding author.

## Abbreviations

The following abbreviations are used in this manuscript:

2D models	Bi-dimensional Models
3D models	Three-dimensional Models
ASCs	Adult Stem Cells
BSA	Bovine Serum Albumin
CCD detector	Charge-Coupled Device Detector
CCK8	Cell Counting Kit-8 Assay
CD105	Endoglin
DAPI	4′,6-Diamidino-2-Phenylindole
DMEM-LG	Dulbecco’s Modified Eagle’s Medium with Low Glucose
DMSO	Dimethyl Sulfoxide
DPSCs	Dental Pulp Stem Cells
EC	Endothelial cells
EDTA	Ethylenediaminetetraacetic Acid
FBS	Fetal Bovine Serum
FITC	Fluorescein Isothiocyanate
FWHM	Full Width at Half Maximum
HA	Hydroxyapatite
hDP-ECM	Human Dental Pulp derived ExtraCellular Matrix
MSCs	Mesenchymal Stem/Stromal Cells
OCT	Optimal Cutting Temperature
ODM	Osteogenic Differentiation Medium
OSX	Osterix
PBS	Phosphate-Buffered Saline
PFA	Paraformaldehyde
RMS	Raman microspectroscopy
RT	Room Temperature
RT-qPCR	Reverse Transcription quantitative Polymerase Chain Reaction
RUNX2	Runt-related Transcription Factor 2
SD	Standard Deviation
